# Performance-Related Physiological and Haematological Changes During Pregnancy and Postpartum in a Well-Trained Cyclist Performing Endurance Training

**DOI:** 10.3389/fphys.2022.762950

**Published:** 2022-05-09

**Authors:** Nicki Winfield Almquist, Øyvind Sandbakk, Guro Strøm Solli

**Affiliations:** ^1^ Section for Health and Exercise Physiology, Inland Norway University of Applied Sciences, Lillehammer, Norway; ^2^ Department of Nutrition, Exercise and Sports, The August Krogh Section for Molecular Physiology, Faculty of Science, University of Copenhagen, Copenhagen, Denmark; ^3^ Centre for Elite Sports Research, Department of Neuromedicine and Movement Science, Norwegian University of Science and Technology, Trondheim, Norway; ^4^ Faculty of Health Sciences, School of Sport Sciences, UiT The Arctic University of Norway, Tromsø, Norway; ^5^ Department of Sports Sciences and Physical Education, Nord University, Bodø, Norway

**Keywords:** structured endurance training, gestation, haemoglobin mass, endurance performance, trimester, cycling

## Abstract

**Purpose:** To describe the performance-related physiological and haematological changes in a well-trained cyclist (peak oxygen uptake, VO_2peak_: 54.9 ml min^−1^·kg^−1^) performing endurance training during pregnancy and postpartum.

**Methods:** Training data was systemized by training form (endurance and resistance), intensity (low- (LIT), moderate-, and high-intensity training), and modality (cycling, running, hiking, XC-skiing, strength training and other). Power output at 4 mmol L^−1^ [BLa^−^] (L_4_), maximal aerobic power (W_max_), and VO_2peak_ as well as haemoglobin mass, blood volume, plasma volume and red blood cell volume (RBCV) were measured at different time points during pregnancy and 12 weeks postpartum.

**Results:** L_4_ and W_max_ increased by 3% while absolute VO_2peak_ was unaltered from gestational wk 2 to 14, despite 12 and 14% increases in RBCV and BV. After delivery, BV was reduced by 7% but RBCV was maintained 5% above start-pregnancy levels, while VO_2peak_ almost returned to (-1%), and W_max_ increased by 5% above start-pregnancy levels 12 weeks postpartum.

**Conclusion:** This case-study illustrates a disassociation between increases in haematological values and VO_2peak_ during pregnancy. Furthermore, a quick resumption of LIT and a gradually increasing intensity of training in the 12 weeks following delivery ensured a return to start-pregnancy levels of VO_2peak_ and corresponding improvements in W_max_. Although general recommendations cannot be given on the basis of these data, this study provides a framework for investigating pregnant endurance athletes and contributes to the generation of new hypotheses in this field.

## Introduction

Many female endurance athletes train relatively large volumes of endurance training during pregnancy, aiming to maintain physical fitness and rapidly regain their performance-level after delivery. Previous studies indicate that well-trained women can benefit from low- (LIT) to moderate-intensity (MIT) endurance training during an uncomplicated pregnancy, thereby facilitating a rapid return to competitive sport (Kardel, 2005). However, the employment of high-intensity training (HIT) during pregnancy is more controversial (Salvesen et al., 2012), and the effects of systematic endurance training and pregnancy-induced hematological alterations on endurance performance have not been described coherently during and after pregnancy in well-trained athletes.

The pregnant athlete undergoes similar physiological changes as other pregnant women, including major cardiovascular changes from the fifth week of gestation (Duvekot et al., 1993; Morris et al., 2015). To ensure sufficient blood supply to the growing foetus, these changes include reduced vascular resistance, increased ventilation, heart rate (HR) and cardiac output as well as a progressive rise in maternal blood volume (Pivarnik et al., 1994; Ekholm and Erkkola, 1996; Gilson et al., 1997). After giving birth, these cardiovascular and haematological changes seem to gradually return to normal within 6–12 weeks (Clapp and Capeless, 1997). However, it is currently unknown how these cardiovascular changes together with high volumes of endurance training influence performance-related physiological variables during pregnancy and postpartum.

One of the major challenges of doing research on pregnant athletes is recruiting a suffient number of participants. Case studies are therefore valuable tools to gather information about physiological changes and the possible effects of training occurring during pregnancy and postpartum. A recent study reported that the world’s most successful cross-country skier was able to maintain a high training volume during most of her pregnancy, with a gradual reduction of training volume during the third trimester (Solli and Sandbakk, 2018). Her oxygen uptake (*V*O_2_) at the estimated lactate threshold decreased to 95% of absolute pre-pregnancy values in the first trimester and further to 93% in the second trimester. After delivery, a relatively rapid return to pre-pregnancy levels was observed, with the *V*O_2_ at the lactate threshold being back at pre-pregnancy levels in week 6–12 and above (103%) in week 13–21 postpartum. However, no data on the accompanied development of haematological variables were measured. To better understand the underlying mechanisms of the physiological changes during pregnancy and postpartum in well-trained women, data including training characteristics, performance-related and haematological variables are needed.

During a previous training intervention (Almquist et al., 2022), in which the effects of periodized endurance training (cycling; 74%, XC-skiing/running; 24%, resistance training 2% of the sessions) were investigated, one of the female participants became pregnant during the intervention period. This participant wanted to complete the training intervention (see ethical considerations discussed below), which provided a unique data set on the haematological and physiological changes occurring during pregnancy and postpartum in a well-trained female cyclist in relation to maintained endurance exercise training. Therefore, this study used a single case approach to describe the haematological and physiological changes in a well-trained cyclist performing semi-structured endurance training during pregnancy and postpartum.

## Materials and Methods

### Participant

The participant was a two-child mother, 37.3 years of age, 177 cm body height and 65.7 kg body mass, and a well-trained cyclist, according to the classification by (Decroix et al., 2016). As recorded 5 weeks prior to the start of the intervention, she exercised regularly (4 ± 1 sessions·week^−1^) including endurance and resistance training (4.2–6.9 h week^−1^ in late December) and competed in amateur-level cycling competitions. During her pregnancy, she also consumed iron supplementation according to general guidelines for pregnant women.

### Ethical Considerations

Prior to the training intervention, the participant had received written and oral information about potential risks and discomforts associated with the training and testing and had given her written consent to participate. When the participant found out that she was pregnant, she was made aware of the potential risk factors when exercising during pregnancy and again informed that she could withdraw from the study. Since the participant still wanted to complete the training intervention, she was allowed by the research team to continue, but was instructed to avoid pushing herself more than what she felt comfortable with. The participant was in her third pregnancy and did not experience any discomfort or other problems during or after the pregnancy. She had a natural birth without complications and bore a healthy child of 49 cm and 3,238 g. Based on this, the publication of this case study was evaluated to be ethically sound by the Regional Ethics Committee (Ref. 231,427) since the collected data can contribute with new knowledge about changes in well-trained women’s physiology in response to endurance training during pregnancy and postpartum.

### Exercise Training

**FIGURE 1:**
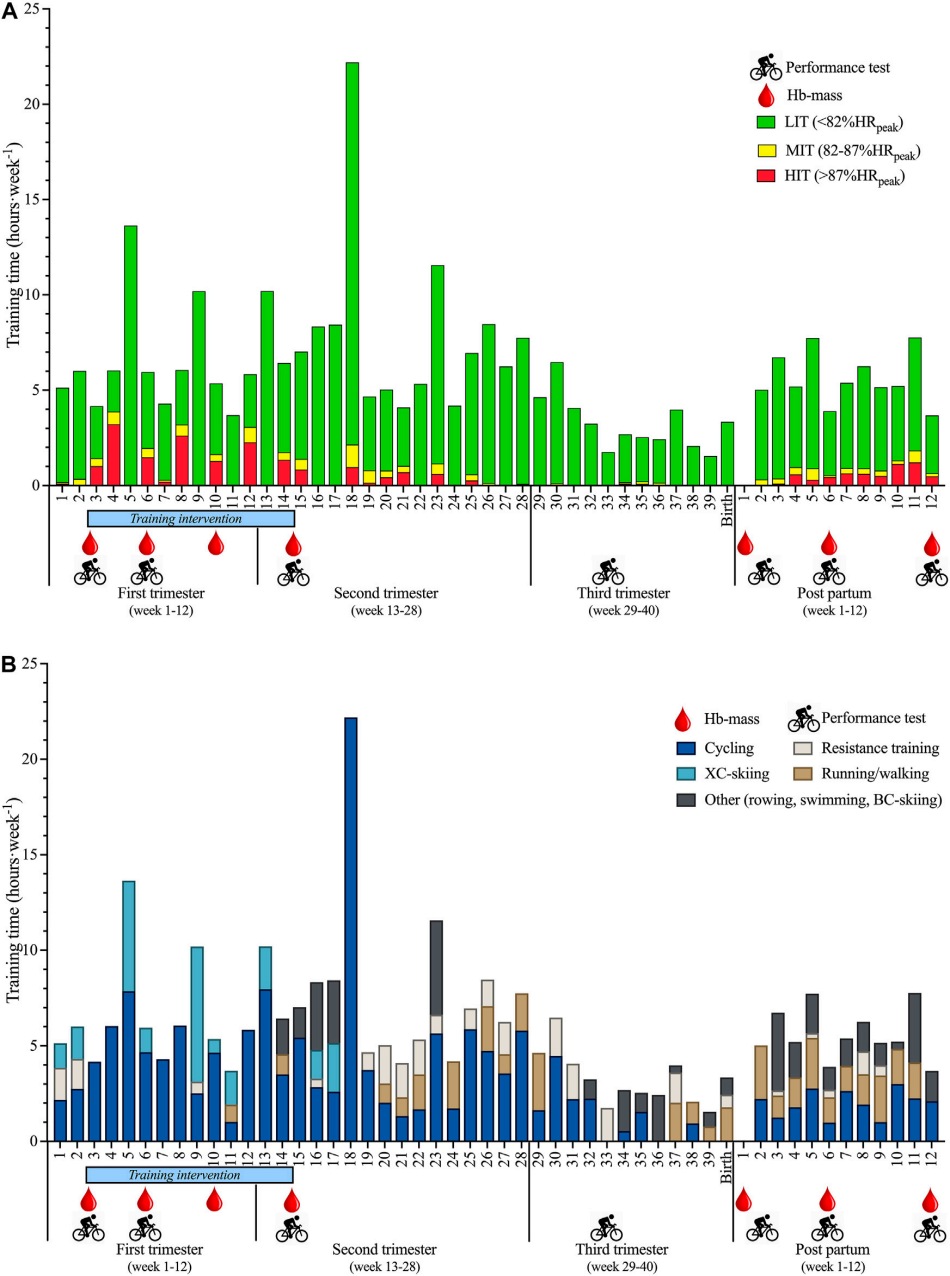
**(A)** Schematic overview of experimental testing and exercise training distribution in three intensities (LIT, low-intensity; MIT, moderate-intensity; HIT, high-intensity training) as described previously (Seiler, 2010), and **(B)** exercise modalities before-, during pregnancy, and 12 weeks postpartum.

All training was recorded from the start of pregnancy and during the following 52 weeks. The start of the block-periodized training intervention took place in the third week of the participant’s first trimester (Figure 1). As part of the training intervention, she completed 12 weeks of structured cycling training as previously described (Almquist et al., 2022). The structured training consisted of; four moderate-intensity interval training sessions (4 × 12 min) in week 1, three to six low-intensity training sessions (>60 min continuous cycling) in week 2, and four high-intensity interval training sessions (5 × 5 min) in week 3. The fourth week of each meso cycle was prescribed as a recovery week. This 4-week mesocycle was repeated three times, including a total of 12 weeks of structured block-periodized training. All sessions were carried out using an effort-based approach and the participant was instructed to perform all sessions at the highest possible average power output that she felt comfortable with. Hence, the majority of the HIT-time during the training intervention was spent in the lower part of HIT-zone (8.3 h at 87–92%HR_peak_) compared to the higher part (6.0 h > 93%HR_peak_). Then, until giving birth, the participant adjusted her training (intensity and volume) according to her own preferences. After delivery, the participant quickly took up her habitual training routines on her own initiative, and gradually increased training volume and intensity in the following weeks. The first HIT-sessions were re-introduced in the third week postpartum.

### Measurements

#### Training Data

Training data were recorded daily by the participant in a digital diary (Trainingpeaks.com, Colorado, United States), including information about intensity and exercise modality (cycling, running/hiking, XC-skiing, strength training and other). The endurance training was registered in three intensity zones based on actual HR registration as previously described (Seiler, 2010); LIT (<82%HR_peak_), MIT (82–87%HR_peak_), and HIT (>87%HR_peak_), as well as comments regarding session details. A total of 134 LIT-sessions, 30 MIT-sessions, 43 HIT-sessions, and 52 resistance training sessions were performed during a year representing 52/11/17/20% of total sessions, respectively, averaging 4.6 ± 2.0 sessions per week. Average training volume was maintained at 6.7 ± 2.9 and 7.9 ± 4.8 h week^−1^ during the first and second trimesters, respectively. The number of LIT, MIT, HIT, and resistance exercise sessions represented 48/25/20/8% during the first trimester and 56/8/15/20% during the second trimester, respectively. During the third trimester, the training volume decreased to 3.8 ± 2.0 h week^−1^ (Figure 1) and included primarily LIT and resistance training (67/7/2/24%). After delivery, the training volume increased from 4.6 ± 2.5 h week^−1^ during the first 6 weeks postpartum to 5.6 ± 1.4 h week^−1^ during week 6–12, while the intensity of exercise gradually increased. Likewise, the distribution of sessions performed as LIT/MIT/HIT/resistance training changed from 61/12/17/46% in the first 6 weeks to 50/5/37/45% in week 6–12, respectively.

#### Physiological Measurements

The physiological measurements were performed during gestational weeks 2, 6, 10, 14 and 33, in addition to weeks 2, 6 and 12 weeks postpartum (Figure 1). This included measurements of power output at 4 mmol L^−1^ [BLa^−^] (L_4_), maximal aerobic power (W_max_) and peak oxygen uptake (VO_2peak_). The percentage typical error (%TE) for L_4_, W_max_, and VO_2peak_ are 1.9%, 1.0%, and 3.1% (*unpublished data from our lab*). Therefore, alterations larger than the %TE are described as changes in the results section. All protocols and equipment used are described in detail here (Almquist et al., 2020). Cycling tests were performed on an electromagnetically braked cycle ergometer (Lode Excalibur Sport, Lode B. V. Groningen, Netherlands) and VO_2_ was measured using a computerized metabolic system with a mixing chamber (Oxycon Pro, Erich Jaeger, Hoechberg, Germany). The participant was instructed to perform until volitional exhaustion and to avoid performing beyond her perceived tolerance during the VO_2peak_ ramp test and reached [BLa^−^] ≥8.9 mmol L^−1^, RER ≥1.12, and an RPE of 20. VO_2peak_ was calculated as the highest average of a 1-min moving average. Peak heart rate (HR_peak_) was registered as the highest achieved HR during the measurement of VO_2peak_. W_max_ was calculated as the mean power output during the last minute of the incremental test. Haemotological measurements were performed in gestational weeks 2, 6, 10 and 14 in addition to weeks 1, 6 and 12 postpartum. This included measurements of haemoglobin mass (Hb-mass), blood volume (BV), plasma volume (PV) and red blood cell volume (RBCV) on an OpCO (Detalo Performance, Detalo Health, Birkerød, Denmark) using a modified version of the carbon monoxide (CO) rebreathing technique (Siebenmann et al., 2012), as previously described (Almquist et al., 2021). The %TE for HB-mass measurements is ∼2% as previously reported from our lab (Ronnestad et al., 2021). These measurements were performed on four occasions during the participant’s first trimester (weeks 2, 6, 10, and 14 according to the training intervention) and at weeks 6 and 12 postpartum. In the general population living in metropolitan cities, CO bound to hemoglobin (COHb%) is ∼2% for non-smokers and 10–15% for smokers (Gozubuyuk et al., 2017). Pregnant women are considered CO-poisoned at COHb% >15% (Gozubuyuk et al., 2017). Therefore, in the present case study, low volumes of CO (1 ml kg^−1^ body weight) were applied and the maximum COHb% was 8.4%, which was in the third week of pregnancy. The mean HbCO% for all measurements performed on the participant was 7.2 ± 0.5.

## Results

### Body Mass

Body mass increased gradually during pregnancy, peaking at 85.0 kg during gestational week 40, which was 19.3 kg above the start-pregnancy level (week 2). After delivery, body mass was quickly reduced, being 3.6 kg above start-pregnancy level 12 weeks postpartum. Consequently, performance-related measures expressed relative to body mass were generally reduced during pregnancy but returned towards start-pregnancy level 12 weeks postpartum.

### Changes During Pregnancy

An overview of the physiological and haemotological measurements from the beginning of pregnancy (week 2) to 12 weeks postpartum is presented in Table 1.

**TABLE 1:** Haematological-, and performance-related measures from the beginning of pregnancy (week 2) to 12 weeks (wk+12) postpartum.

	Pregnancy					Postpartum				Changes from Gestational wk 2 (%)							
	wk	wk	wk	wk	wk	wk	wk	wk	wk	wk	wk	wk	wk	wk	wk	wk	wk
	2	6	10	14	33	+1	+2	+6	+12	6	10	14	33	+1	+2	+6	+12
Body mass (kg)	65.7	67.2	67.2	70.9	80.4	77.8	73.6	72.8	69.3	2.3	2.3	7.9	22.4	18.4	-	10.8	5.5
Hb-mass (g)	595	635	726	696	-	755	-	620	632	6.7	22.0	17.0	-	26.9	-	4.2	6.2
RBCV (ml)	1857	1952	2,300	2083	-	2,295	-	1938	1946	5.1	23.9	12.2	-	23.6	-	4.4	4.8
BV (ml)	4,686	4,790	6,154	5,341	-	4,884	-	4,171	4,374	2.2	31.3	14.0	-	4.2	-	-11.0	-6.7
PV (ml)	2,829	2,838	3,854	3,258	-	2,588	-	2,232	2,427	0.3	36.2	15.2	-	-8.5	-	-21.1	-14.2
Hb-conc. (g·dl^−1^)	12.7	13.3	11.8	13	-	15.5	-	14.9	14.5	4.7	-7.1	2.4	-	22.0	-	17.3	14.2
Haematocrit (%)	39.6	40.8	37.4	39	-	47	-	46.5	44.5	3.0	-5.6	-1.5	-	18.7	-	17.4	12.4
W_max_ (W)	300	300	-	309	-	-	-	301	316	0.0	-	3.0	-	-	-	0.3	5.3
L_4_ (W)	182	192	-	188	180	-	151	177	178	5.5	-	3.3	-1.1	-	-17.0	-2.7	-2.2
VO_2peak_ (mL·min^−1^)	3,606	3,453	-	3,637	-	-	-	3,352	3,566	-4.2	-	0.9	-	-	-	-7.0	-1.1
Hb-mass (g·kg^−1^)	9.1	9.5	10.8	9.8	-	9.7	-	8.5	9.1	4.4	18.7	7.7	-	6.6	-	-6.6	0.0
RBCV (ml·kg^−1^)	28.3	29.1	34.2	29.3	-	29.5	-	26.6	28.1	2.8	20.8	3.5	-	4.2	-	-6.0	-0.7
BV (ml·kg^−1^)	71.3	71.5	91.6	75.2	-	62.8	-	57.3	63.1	0.3	28.5	5.5	-	-11.9	-	-19.6	-11.5
PV (ml·kg^−1^)	43.1	42.4	57.3	45.9	-	33.3	-	30.7	35.0	-1.6	32.9	6.5	-	-22.7	-	-28.8	-18.8
W_max_ (W·kg^−1^)	4.6	4.5	-	4.4	-	-	-	4.1	4.6	-2.2	-	-4.3	-	-	-	-10.9	0.0
VO_2peak_ (ml·kg^−1^·min^−1^)	54.9	51.4	-	51.3	-	-	-	46.0	51.5	-6.4	-	-6.6	-	-	-	-16.2	-6.2
L_4_ (W·kg^−1^)	2.8	2.9	-	2.6	2.2	-	2.1	2.4	2.6	3.6	-	-7.1	-21.4	-	-25.0	-14.3	-7.1

Hb-mass, Hemoglobin mass; RBCV, red blood cell volume; BV, blood volume; PV, plasma volume; W_max_, Maximal aerobic power; VO_2peak_, Peak oxygen uptake; L_4_, Power output at 4 mmol L^−1^ [BLa^−^].

W_max_ was unchanged in week 6, before it increased by 3% (+9 W) in week 14 following 12 weeks of structured endurance training. In contrast, L_4_ increased by 6% (+10 W) in week 6, and was 3% (+6 W) above start pregnancy levels in week 14 following the structured endurance training period. L_4_ was maintained at start-pregnancy level (-2W) in week 33 after a period of mainly LIT. Absolute VO_2peak_ was reduced by 4% (-153 ml min^−1^) in gestational wk 6, before returning to 1% (+31 ml) above start-pregnancy values in week 14. Haematological variables, including RBCV and Hb-mass increased by 5% (+95 ml) and 7% (+40 g) in week 6 and further increased to 14% (+226 ml) and 17% (+101 g) above start pregnancy levels in week 14, respectively.

### Changes During Postpartum

W_max_ returned to start-pregnancy levels in week 6 and further increased by 5% (+16 W) above the start-pregnancy value in wk 12. Two weeks postpartum, L_4_ was reduced by 17% (-31 W) compared to start-pregnancy level, before quickly increasing to values being only 2–3% (4–5 W) below start-pregnancy level in week 6 and 12 postpartum. Absolute VO_2peak_ was reduced by 7% (-254 ml) compared to the start-pregnancy level and returned to 1% (−40 ml) below the start-pregnancy level in week 12 postpartum.

The highest measurements of RBCV and Hb-mass occurred 1 week postpartum with values being 24% (+438 ml) and 27% (+160 g) above start-pregnancy levels, respectively, remaining ∼4–6% (81–89 ml and 25–37 g, respectively) above start-pregnancy levels in wk 6 and 12 weeks postpartum. However, substantial decreases in PV were observed 1, 6, and 12 weeks postpartum (−241 ml, −597 ml, and −402 ml, respectively), coinciding vastly increasing haematocrit (+7.4, +6.9, and +4.9 %-points, respectively) compared to start-pregnancy.

## Discussion

This case-study describes performance-related physiological and haematological changes during pregnancy and postpartum for a well-trained cyclist performing endurance training. From gestational week 2 to 14, W_max_ and L_4_ were improved by 3% while the absolute VO_2peak_ was unaltered, despite substantial increases in both RBCV (12%) and BV (14%). Twelve weeks postpartum, W_max_ was 5% above-, while VO_2peak_ was almost back at start-pregnancy levels (−1%). In addition, blood volume was reduced by 7%, while RBCV was maintained 5% above start-pregnancy values.

While HIT is generally regarded as an effective cardiovascular stimulus (Laursen and Jenkins, 2002; Buchheit and Laursen, 2013), the unchanged absolute values of VO_2peak_, despite substantial amounts of HIT and vastly increased RBCV and BV during the initial phase of pregnancy, could indicate that HIT does not provide additional benefits above the otherwise recommended MIT during pregnancy (Mottola et al., 2018). The present findings are in line with a previous study where the authors investigated pregnant athletes, reporting no increase in VO_2peak_ during pregnancy but substantially improved VO_2peak_ postpartum (Kardel, 2005). Hence, it could be speculated that the lack of VO_2peak_ improvement, despite increased RBCV, BV, and Hb-mass, is due to a protective mechanism for the growing foetus, since a clear relation between Hb-mass and VO_2peak_ is evident in non-pregnant athletes (Lundby and Robach, 2015). However, the mechanisms underlying this dissociation between changes in haematological measures and VO_2peak_ need further investigation. Although adverse effects of HIT on fetal well-being are not consistently reported (Szymanski and Satin, 2012; Anderson et al., 2021), the recommendation of MIT rather than HIT during pregnancy would reduce any risk of fetal bradycardia previously observed with maternal exercise intensities above 90% HR_peak_ (Salvesen et al., 2012). The observed haematological alterations are in line with previous reviews in pregnant athletes (Bo et al., 2016). However, haemodilution as normally occurs during pregnancy (Pivarnik et al., 1994), was only observed in week 10 in our participant. The haematocrit, otherwise, remained ∼39–41%, whether this relates to hydration, exercise, or error of measurements is speculative.

Interestingly, RBCV and Hb-mass remained 5–6% higher than start-pregnancy values during wk 6 to 12 postpartum, while haematocrit was 7 and 5 %-points higher, respectively, due to a rapid decrease in PV postpartum. These haematological alterations were followed by a 5 and 6%-point increase in W_max_ and VO_2peak_, which is higher than the reported %TE for these measurements. Together, this could indicate that pregnancy-related alterations in haematological measures might potentiate subsequent beneficial training adaptations during the postpartum period. Although our participant’s PV, BV and body mass were not returned to start-pregnancy values in wk 12 postpartum, the progress of these parameters together with the regained absolute VO_2peak_ and improved W_max_ suggest a rapid return to start-pregnancy levels, when a reasonable progression in training volume and intensity is ensured. Indicatively, L_4_ decreased after a long period of reduced training volume and intensity, despite a greater dilution of blood lactate in the 4% higher BV, but quickly improved with increased volume and intensity. This is in line with a previous study (Solli and Sandbakk, 2018), where VO_2_ at the estimated lactate threshold rapidly increased up to- (wk 6–12), and above (wk 13–20) pre-pregnancy values during the postpartum period in a successful Olympic athlete. Overall, this case study suggests some lingering hematological benefits and underlines the positive effect of a quick resumption of LIT with a gradually increasing intensity of training in the first 12 weeks after delivery. Together, this highlights the necessity of further investigations of the associations between endurance training at different intensities, haematological alterations, VO_2peak_, and endurance performance in female athletes during and after pregnancy.

While the previously estimated risk of premature birth and low birth-weight following high volumes of endurance training during pregnancy, has been rejected (Haakstad and Bo, 2011; Thangaratinam et al., 2012; Di Mascio et al., 2016), findings indicate an increased abortion risk with very high training volumes and/or HIT during the first weeks after conception (Hegaard et al., 2016). In this context, the total training volume performed during pregnancy in the current case is clearly lower than in a previous study (Solli and Sandbakk, 2018) but showed the same relative reductions in training volume throughout pregnancy as previous studies (Potteiger et al., 1993; Bailey et al., 1998; Solli and Sandbakk, 2018; Sundgot-Borgen et al., 2019). In contrast to these studies, our participant also conducted relatively large amounts of HIT during the first trimester. This can be seen as controversial as a previous study has indicated that fetal wellbeing may be compromised during HIT (>90% HR_peak_) among pregnant athletes (Salvesen et al., 2012). However, other studies report overall fetal wellbeing as reassuring after short-duration, strenuous exercise in both active (Anderson et al., 2021) and inactive pregnant women (Szymanski and Satin, 2012). Furthermore, elite athletes exercising >8–10 h wk^−1^ during pregnancy have not been reported to be at higher risk of complications during delivery or to affect the child negatively compared to non-exercising women (Sigurdardottir et al., 2019). Most importantly, the present participant bore a healthy child and no adverse effects of maintaining a high amount of HIT during the first trimester were found. However, the current recommendation of MIT rather than HIT during pregnancy seems reasonable, as it likely induces satisfactory physiological adaptations while avoiding any possible risks associated with performing HIT during pregnancy. In this participant, a gradual increase from 17% HIT sessions during the first 6 weeks postpartum, to 37% HIT sessions in the weeks 6–12 post partum, may have been beneficial since VO_2peak_, and W_max_ increased above start-pregnancy levels.

## Conclusion

Despite structured endurance training and substantial increases in both red blood cell volume and blood volume, no increase in absolute VO_2peak_ was observed from gestational week 2 to 14 in this case-study of a well-trained cyclist. Although the training intensity distribution was changed, and included less HIT and relatively more LIT, power output at 4 mmol L^−1^ [BLa^−^] was maintained until gestational week 33. After delivery, red blood cell volume remained 5% above start-pregnancy levels and VO_2peak_ increased by 6%-points when HIT was re-introduced, with a concomitant 5% increase in W_max_ from week 6 to 12 postpartum. Accordingly, this illustrates a disassociation between haematological values and VO_2peak_ during pregnancy and a quick improvement of endurance performance after delivery when gradually returning to start-pregnancy training levels.

Although the methodology used in this study is not suitable for establishing cause-and-effect relationships, the study highlights the necessity of further investigating the associations between endurance training at different intensities, haematological measures, VO_2peak_, and endurance performance in female athletes during and after pregnancy. Furthermore, the study can be used as a framework for future studies investigating pregnant endurance athletes.

## Data Availability

The raw data supporting the conclusion of this article will be made available by the authors, without undue reservation.
